# Inhibition of sulfur mustard-induced cytotoxicity and inflammation by the macrolide antibiotic roxithromycin in human respiratory epithelial cells

**DOI:** 10.1186/1471-2121-8-17

**Published:** 2007-05-24

**Authors:** Xiugong Gao, Radharaman Ray, Yan Xiao, Peter E Barker, Prabhati Ray

**Affiliations:** 1 Section of Molecular Biology, Department of Biology, Division of Experimental Therapeutics, Walter Reed Army Institute of Research, Silver Spring, MD 20910, USA; 2 Cell and Molecular Biology Branch, Research Division, US Army Medical Research Institute of Chemical Defense, Aberdeen Proving Ground, MD 21010, USA; 3 DNA Measurements Group, Chemical Science and Technology Laboratory, National Institute of Standards and Technology, Gaithersburg, MD 20899, USA

## Abstract

**Background:**

Sulfur mustard (SM) is a potent chemical vesicant warfare agent that remains a significant military and civilian threat. Inhalation of SM gas causes airway inflammation and injury. In recent years, there has been increasing evidence of the effectiveness of macrolide antibiotics in treating chronic airway inflammatory diseases. In this study, the anti-cytotoxic and anti-inflammatory effects of a representative macrolide antibiotic, roxithromycin, were tested *in vitro *using SM-exposed normal human small airway epithelial (SAE) cells and bronchial/tracheal epithelial (BTE) cells. Cell viability, expression of proinflammatory cytokines including interleukin (IL)-1β, IL-6, IL-8 and tumor necrosis factor (TNF), and expression of inducible nitric oxide synthase (iNOS) were examined, since these proinflammatory cytokines/mediators are import indicators of tissue inflammatory responses. We suggest that the influence of roxithromycin on SM-induced inflammatory reaction could play an important therapeutic role in the cytotoxicity exerted by this toxicant.

**Results:**

MTS assay and Calcein AM/ethidium homodimer (EthD-1) fluorescence staining showed that roxithromycin decreased SM cytotoxicity in both SAE and BTE cells. Also, roxithromycin inhibited the SM-stimulated overproduction of the proinflammatory cytokines IL-1β, IL-6, IL-8 and TNF at both the protein level and the mRNA level, as measured by either enzyme-linked immunosorbent assay (ELISA) or real-time RT-PCR. In addition, roxithromycin inhibited the SM-induced overexpression of iNOS, as revealed by immunocytochemical analysis using quantum dots as the fluorophore.

**Conclusion:**

The present study demonstrates that roxithromycin has inhibitory effects on the cytotoxicity and inflammation provoked by SM in human respiratory epithelial cells. The decreased cytotoxicity in roxithromycin-treated cells likely depends on the ability of the macrolide to down-regulate the production of proinflammatory cytokines and/or mediators. The results obtained in this study suggest that macrolide antibiotics may serve as potential vesicant respiratory therapeutics through mechanisms independent of their antibacterial activity.

## Background

2,2'-Dichlorodiethyl sulfide (sulfur mustard, SM) is a vesicant agent that was used as a chemical weapon during World War I and more recently during the Iran-Iraq conflict; therefore, it remains a significant military and civilian threat [1]. Damage due to SM inhalation has been found to be dose-dependent [2,3]. At low to moderate doses, the upper respiratory tract is mostly affected, whereas at higher doses, damage is seen in the lower lung, including the alveoli. SM-induced epithelial cell injury and cell death in the tracheobronchial tree leads to inflammation and sloughing of the mucosa during acute stages. The clinical signs of SM inhalation include asthma, chronic bronchitis, bronchiectasis, and pulmonary fibrosis [4]. Pulmonary damage and associated secondary infections have been responsible for most fatalities [3]. At present, there is no effective therapy for the victims due to lack of understanding of the pathophysiological processes of SM inhalation injury.

SM acts as an electrophile that alkylates cellular and extracellular components of living tissue. As a result, complex cellular events develop, including cell cycle arrest, the synthesis and release of inflammatory mediators, and cytotoxicity. Following these cellular effects are tissue responses such as inflammation and tissue damage [5]. The cause of the acute injury appears to be the premature, sudden and massive release of destructive enzymes and mediators of inflammation such as proinflammatory cytokines, including interleukin (IL)-1β, IL-6, IL-8 and tumor necrosis factor (TNF), and inducible nitric oxide synthase (iNOS).

Proinflammatory cytokines are essential mediators of cell-to-cell signals in physiological and pathological immune responses and in the inflammatory response. Under normal conditions, these cytokines act as crucial signals in the development of appropriate defenses. However, exaggerated or prolonged release can lead to pathological conditions [6].

iNOS is the inducible isoform of nitric oxide synthase, the enzyme that catalyzes the synthesis of nitric oxide (NO), a short-lived free radical gas and a pleiotropic mediator involved in the regulation of vascular smooth muscle tone and proliferation, cell-mediated immunity, and inflammation [7,8]. NO also plays a vital beneficial role in wound healing through its functional influences on angiogenesis, inflammation, cell proliferation, matrix deposition, and remodeling (for review see [9]). However, NO can exert deleterious effects when it is inappropriately generated or overproduced, and excessive amounts of NO and its metabolites, such as peroxynitrite, may contribute to the pathophysiology of inflammation and the resultant tissue damage [10-12]. Production of NO is upregulated in a variety of inflammatory diseases in which iNOS may be involved [13,14].

Macrolides are a group of antibiotics that were initially discovered because of their antibacterial properties. The name "macrolide" is derived from their structure, which is characterized by a macrocyclic lactone ring with various amino sugars attached [15]. Roxithromycin is one of the commonly used macrolides approved by the U.S. Food and Drug Administration (FDA) [16]. In recent years, there has been increasing evidence of the effectiveness of macrolide antibiotics in treating chronic airway inflammatory diseases through mechanisms distinct from their antibacterial activity (for review see [17]). Although the mechanisms underlying this effect are still unclear, macrolides have been shown to affect several pathways of the inflammatory process, including the migration of neutrophils, the oxidative burst in phagocytes, and the production of proinflammatory cytokines [18]. In this study, the anti-cytotoxic and anti-inflammatory effects of roxithromycin were tested using normal human small airway epithelial (SAE) cells and bronchial/tracheal epithelial (BTE) cells exposed to SM as *in vitro *models. Cell viability and expression of proinflammatory cytokines and iNOS were examined.

## Results

### Cytotoxicity

**Figure 1:**
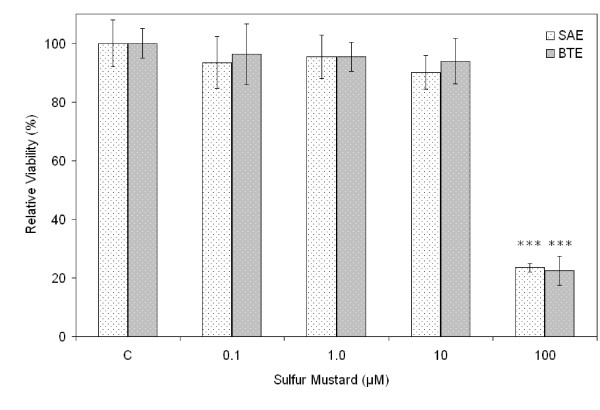
**Cytotoxicity of SM on SAE and BTE cells**. Cells were exposed to various concentrations of SM. Cell viability was measured using the MTS assay 24 h after exposure, and is expressed as a percentage of the control (unexposed cells, "C"). ***P < 0.001 versus control.

**Figure 2:**
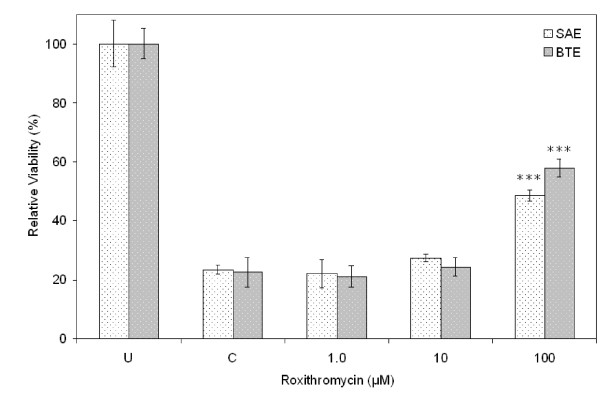
**Effect of roxithromycin on SM cytotoxicity in SAE and BTE cells**. Various concentrations of roxithromycin were added to cells exposed to 100 μM SM. Cell viability was measured using the MTS assay 24 h after exposure, and is expressed as a percentage of the value obtained from cells unexposed to SM ("U"). ***P < 0.001 versus control (cells exposed to SM without roxithromycin, "C").

The MTS assay showed that SM exerted cytotoxicity in a concentration-dependent manner in SAE and BTE cells. At 10 μM, no significant decrease in cell viability was observed. At 100 μM, however, SM caused severe toxicity: the viability of both cell types decreased to ~20% compared with untreated cells 24 h after exposure (Fig. 1). In contrast, roxithromycin reduced SM cytotoxicity in SAE and BTE cells; incubation with 100 μM roxithromycin increased the cell viability from ~20% to ~50% and ~60%for SAE and BTE cells, respectively, 24 h after exposure to 100 μM SM (Fig. 2). No significant protective effects of roxithromycin were observed at concentrations lower than 10 μM. Roxithromycin alone had no effects on cell viability of SAE and BTE cells at the concentration range tested (0.1 μM – 100 μM, data not shown).

**Figure 3:**
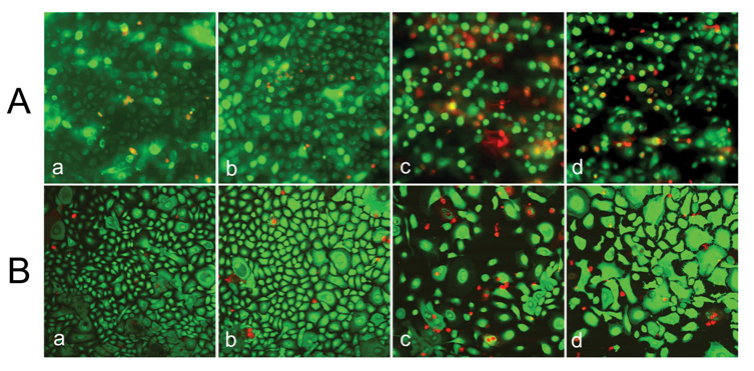
**Anti-cytotoxicity effect of roxithromycin on SM-treated SAE and BTE cells**. Cell viability was examined by Calcein AM/EthD-1 fluorescence staining 24 h after exposure. The images for SAE cells (A) were captured by fluorescence microscopy whereas those for BTE cells (B) by confocal fluorescence microscopy. *a*) control (unexposed), *b*) 100 μM roxithromycin, *c*) 100 μM SM, and *d*) 100 μM SM + 100 μM roxithromycin. Cells stained green were alive and those stained red dead.

Calcein AM/EthD-1 staining showed a similar protective effect of roxithromycin on SM-exposed SAE and BTE cells (Fig. 3). Live cells are distinguished by the presence of ubiquitous intracellular esterase activity, which converts the virtually nonfluorescent cell-permeant calcein AM to the intensely fluorescent calcein, producing an intense uniform green fluorescence in live cells. Dead cells usually are characterized by damaged membranes. EthD-1 enters dead cells and undergoes a 40-fold enhancement of fluorescence upon binding to nucleic acids, thereby producing a bright red fluorescence in dead cells. EthD-1 is excluded by the intact plasma membrane of live cells [19]. It is apparent that exposure to 100 μM SM caused significant cell death compared to control (unexposed) cells, whereas the number of live cells increased substantially when 100 μM roxithromycin was included. Roxithromycin (100 μM) alone had no effect on cell viability. It was noted that a significant number of cells died shortly after SM exposure and thus detached from the slide, therefore the observed cell density in samples treated with SM was significantly lower than that of untreated samples. Also, a large number of BTE cells enlarged after treatment with SM and roxithromycin (Fig. 3). The reason for this morphological change was unknown. Similar morphology change was not observed in SAE cells.

Previous studies have implied that inflammatory cytokines or mediators are involved in SM-induced injuries and cell death. For example, suppressed expression of the proinflammatory cytokines IL-8 and IL-6 in human epidermal keratinocytes led to increased viability of SM-treated cells [20]. In order to understand the mechanism of the protective effect of roxithromycin and verify if the decreased cytotoxicity is due, at least in part, to a reduced inflammation, we further evaluated the effect of roxithromycin on the expression of some inflammatory mediators, including proinflammatory cytokines and iNOS, at the mRNA and/or protein level.

### Cytokine protein expression

SM stimulated release of proinflammatory cytokines (IL-1β, IL-6, IL-8 and TNF) from SAE and BTE cells into the culture medium, albeit the extent of stimulation varied among different cytokines. A time-course study of cytokine release from cells exposed to ≤ 100 μM SM indicated that, for both cell types, accumulation of all the cytokines reached maximum levels 24 h after exposure and remained stable for at least 48 h (data not shown). Therefore, in the following experiments all the cytokines were measured 24 h post-exposure. In general, it was found that SM exposure induced proinflammatory cytokine release in a concentration-dependent manner, and roxithromycin inhibited the cytokine release, also in a concentration-dependent manner. We noticed that the absolute concentrations of cytokines measured in the culture supernatant were not consistent among replicated experiments, probably due to differences in cell number, confluence, age and other unknown factors. However, when the concentration values were normalized relative to control (unexposed) cells, the data became fairly consistent. For this reason, the results are presented as percentages of the control in each experiment.

#### IL-8

**Figure 4:**
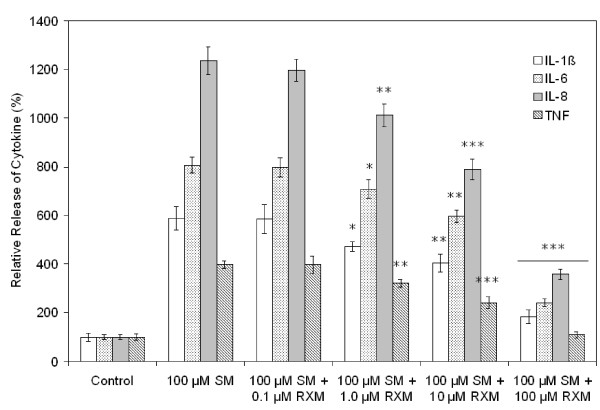
**Inhibition by roxithromycin of cytokine release from SM-exposed SAE cells**. Various concentrations of roxithromycin were added to cells exposed to 100 μM SM. Cytokine release was measured by ELISA 24 h after exposure. Cytokine concentration is expressed as a percentage of the control (unexposed) cells. RXM, roxithromycin. *P < 0.05, **P < 0.01, ***P < 0.001 versus cells exposed to SM only.

Culture medium harvested from control SAE cell cultures showed a gradual accumulation of this cytokine over time that reached a plateau by 24 h. Upon treatment with <10 μM SM, no significant difference compared to the control situation was observed. When treated with 100 μM SM, however, a significantly higher amount of IL-8 was released from SAE cells after 5 h and the accumulation of the cytokine persisted up to 24 h after exposure (data not shown). The final concentration, in the range of 20 ng/ml – 30 ng/ml among experiments, was about 12-fold higher than control cells. When roxithromycin was added to the SAE cell culture medium before SM exposure, concentration-dependent inhibition of the SM-induced cytokine secretion was observed (Fig. 4). Inhibition was observed at a concentration as low as 1 μM and reached 71% when the concentration of roxithromycin was 100 μM. Very similar results were observed for BTE cells, except that the highest concentration of IL-8 released into the medium was in the range of 12–18 ng/ml when cells were exposed to 100 μM SM.

#### IL-6

Similar to IL-8, culture medium harvested from control SAE cell cultures showed a gradual accumulation of IL-6 over time that reached a plateau by 24 h. Upon treatment with <10 μM SM, no significant difference compared to the control situation was observed. When treated with 100 μM SM, however, a significantly higher amount of IL-6 was released from SAE cells after 3 h and accumulation of the cytokine persisted up to 24 h after exposure (data not shown). The final concentration, in the range of 3 ng/ml – 5 ng/ml among experiments, was 8-fold as high as the control cells. When roxithromycin was added to the SAE cell culture medium before SM exposure, concentration-dependent inhibition of SM-induced cytokine secretion was again observed (Fig. 4). The inhibition was observed at a concentration as low as 1 μM and reached 70% when the concentration of roxithromycin was 100 μM. Very similar results were observed for BTE cells, except that the range of the highest concentration of IL-6 released into the medium was 4 ng/ml – 6 ng/ml for cells exposed to 100 μM SM.

#### IL-1β

Unlike IL-6 and IL-8, IL-1β concentration in the culture medium of SAE cells remained low even when the cells were treated with 100 μM SM (<100 pg/ml). Nevertheless, statistically significant concentration-dependent stimulation by SM and concentration-dependent inhibition by roxithromycin were observed. IL-1β concentration in the culture medium increased to 6-fold compared with control cells 24 h after exposure to 100 μM SM (Fig. 4). Inhibition by 100 μM roxithromycin was 69%. Very similar results were observed for BTE cells, except that the highest concentration of IL-1β released into the medium was ~80 pg/ml upon 100 μM SM exposure.

#### TNF

Similar to IL-1β, the concentration of TNF in the culture medium of SAE cells also remained low (<50 pg/ml) even when treated with 100 μM SM. Nevertheless, concentration-dependent stimulation by SM and concentration-dependent inhibition by roxithromycin were observed. TNF concentration in the culture medium increased ~4-fold compared with control cells 24 h after exposure to 100 μM SM and inhibition by 100 μM roxithromycin was 73% (Fig. 4). Very similar results were observed for BTE cells.

### Cytokine mRNA expression

**Figure 5:**
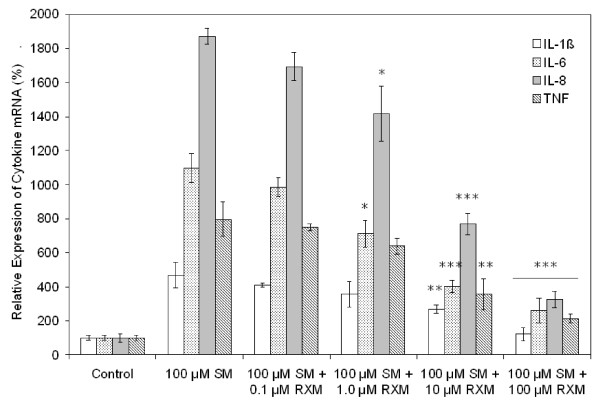
**Inhibition by roxithromycin of cytokine mRNA expression in SM-exposed SAE cells**. Various concentrations of roxithromycin were added to cells exposed to 100 μM SM. Total RNA was isolated from cells 24 h after exposure. Cytokine mRNA was quantified using real-time RT-PCR. Results are expressed as a percentage of the control (unexposed) cells. RXM, roxithromycin. *P < 0.05, **P < 0.01, ***P < 0.001 versus cells exposed to SM only.

The mRNA expression levels of the proinflammatory cytokines in SAE cells were in good agreement with their protein expression patterns obtained from the ELISA experiments. At the mRNA level, SM exposure also induced proinflammatory cytokine expression in a concentration-dependent manner (data not shown). Exposure to 100 μM SM increased cytokine mRNA expression substantially (Fig. 5); for IL-6, IL-8 and TNF, the fold increase was even higher than that of the protein release level measured by ELISA (18-, 12-, and 8-fold vs. 12-, 8-, and 4-fold, respectively). For IL-1β, the increase in its mRNA level was slightly lower than its increase in protein secretion (4-fold vs. 6-fold). Treatment with roxithromycin of the SM-exposed cells decreased the mRNA levels. This inhibitory effect of roxithromycin on mRNA expression was also concentration dependent (Fig. 5). Roxithromycin at 100 μM was effective in reducing the mRNA expression close to normal levels. Similar results were obtained for BTE cells.

### iNOS expression

iNOS is difficult to detect in airway epithelial cells by western blotting or conventional immunocytochemical methods, primarily due to its very low basal level expression. Results from real-time RT-PCR experiments indicated that the iNOS mRNA level was 10^3 ^– 10^5 ^times lower than that of some of the proinflammatory cytokines (data not shown). In this study, we used quantum dots, a new nano-scale material, as the fluorophore in the immunocytochemical detection of iNOS. Because of the high stability, intensity, and signal-to-noise ratio of the fluorescence signal of quantum dots, iNOS was easily detectable in both control (unexposed) and SM-exposed SAE and BTE cells.

**Figure 6:**
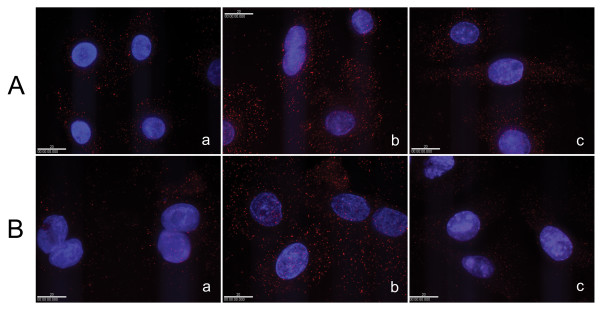
**Effect of roxithromycin on iNOS expression in SM-exposed SAE (A) and BTE (B) cells**. *a*) control; *b*) 100 μM SM; *c*) 100 μM SM + 100 μM roxithromycin. Representative 2D images from each sample are shown. Red spots are signals produced from quantum dots-detected iNOS. The scale bar in each image represents 20 μm.

**Figure 7:**
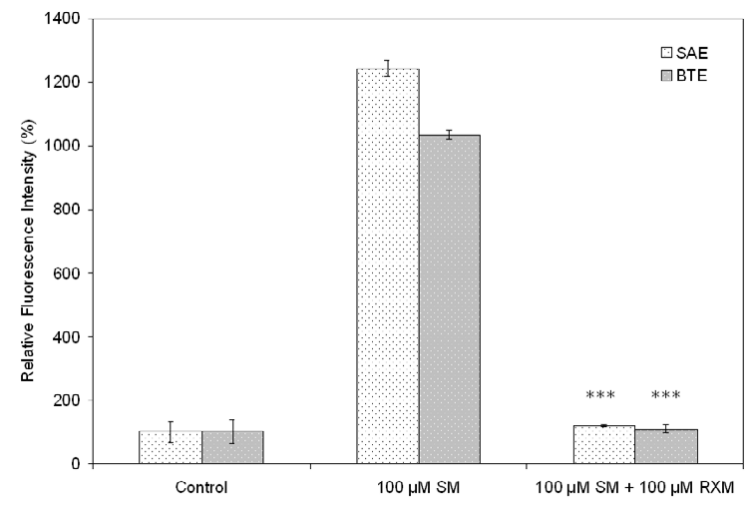
**Relative fluorescence intensity showing quantitatively the effect of roxithromycin on iNOS expression in SM-exposed SAE and BTE cells**. Average fluorescence intensities were computed from cells (n ≥ 10) in each sample and are expressed relative to the control (unexposed cells). RXM, roxithromycin. ***P < 0.001 versus cells exposed to SM only.

Using this new immunocytochemical method we have found that, in both SAE and BTE cells, SM caused overexpression of iNOS, and this effect was inhibited by roxithromycin. Shown in Fig. 6 are immunofluorescence staining images using streptavidin-conjugated quantum dots as detectors. Results from the statistical analysis of the fluorescence signals are shown in Fig. 7. Exposure to 100 μM SM dramatically increased iNOS expression in the two cell types (>10-fold). However, when 100 μM roxithromycin was added to the culture medium before SM exposure, iNOS expression was reduced substantially, to a level comparable to the basal level. Roxithromycin (100 μM) alone had no effect on the signal level detected (data not shown).

## Discussion

SM inhalation causes acute airway inflammation and tissue injury, presumably due to the premature, sudden and massive release of destructive enzymes and mediators of inflammation [3]. Currently there are no effective antidotes for SM-induced inflammation and injury. Macrolide antibiotics have been reported to have immunomodulatory effects and to be effective in treatment of chronic airway inflammation through actions other than their bactericidal activity [17]. In this study, we evaluated the potential of macrolide antibiotics as antidotes for SM-induced injury and inflammation using roxithromycin as a representative macrolide antibiotic for the tests.

Airway inflammation and injury by SM is a complex phenomenon that involves airway epithelial cells, cilia cells, goblet cells and smooth muscle cells interacting with one another under the control of a network of cytokines and other mediators. SAE and BTE cells are considered the first line of defense against inhaled chemicals as they are in direct contact with these chemicals and participate in the pathogenesis of airway injury through the release of oxidants and inflammatory cytokines. It is therefore important to understand the interaction between these cells and the chemical insult, as well as their response to an insult/therapeutic treatment combination such as SM/roxithromycin.

The effect of roxithromycin on SM-induced cell injury was first tested by the MTS cell viability assay and the Calcein AM/EthD-1 fluorescence staining viability assay. The MTS assay showed a concentration-dependent cytotoxicity induced by SM in SAE and BTE cells. For both cell types, 100 μM roxithromycin alleviated the SM cytotoxicity. Calcein AM/EthD-1 fluorescence staining confirmed these results. Together, these results demonstrated the protective effect of roxithromycin against SM-induced injury.

Proinflammatory cytokines play a major role in both acute and chronic inflammatory processes, including those produced by SM. However, these cytokines, particularly when produced in excess, can be pathogenic. Previous studies demonstrated that *in vivo *damage to the skin by SM results in an immunological response defined by increased gene expression of the inflammatory cytokines [6]. In this study we examined the expression of four major inflammatory cytokines, IL-1β, IL-6, IL-8, and TNF. The basal expression levels of the four cytokines were all very low in normal SAE and BTE cells, although IL-6 and IL-8 accumulated continuously in the culture medium from unstimulated cells grown in cell culture. However, exposure of SAE and BTE cells to SM *in vitro *resulted in a rapid increase in the mRNA levels in the cell and the release of the cytokine protein into culture supernatants for all of the four cytokines. Both the mRNA expression and protein release increased several fold in SM-exposed cells relative to unexposed cells for IL-1β and TNF, and increased 10- to 20-fold in the cases of IL-6 and IL-8. Although the final supernatant concentrations for IL-1β and TNF remained low (<100 pg/ml), the concentrations of IL-6 and IL-8, on the other hand, reached very high levels (10 ng/ml – 30 ng/ml). These results suggest that these two inflammatory mediators may play a major role in the mediation of the inflammatory and immune responses initiated by SM inhalation.

In light of the potential role of IL-6 and IL-8 in inflamed airway epithelium, an understanding of the regulation of their production may provide valuable information for treatment of SM exposure. A significant increase in IL-8 release was also reported in human epidermal keratinocytes following SM exposure and has been proposed as a biomarker for SM-induced inflammation [21]. It is also intriguing to note that IL-6 was reported as an anti-inflammatory cytokine in human plasma samples [22] and in human monocytes [23]. Therefore, it is reasonable to speculate that IL-6 is a pleiotropic cytokine whose anti- or pro-inflammatory properties depend on the cell type from which it is produced and to which it is targeted.

iNOS has been suggested to be an important biomarker of inflammation, as its overexpression leads to excessive production of NO, which contributes to the pathophysiology of inflammation and the resultant tissue damage [10]. NO is involved in several types of acute and chronic inflammation [24]. Overproduction of NO by type II NOS or iNOS is associated with the development of airway inflammation [14]. However, the basal level expression of iNOS was low in SAE and BTE cells and therefore was difficult to detect by immunocytochemistry using conventional fluorophores (Texas Red, fluorescein, *etc*.). To improve signal stability and quantitation, an optically stable, new class fluorophore for immunocytochemical detection was employed in this study. Detection of iNOS was based on fluorescence from streptavidin-linked inorganic semiconductor nanocrystals of cadmium selenide [(CdSe)ZnS]. As fluorescence of nanomaterialfluorophores was significantly brighter and more photostable than organic fluorophores such as Texas Red and fluorescein [25,26], we were able to detect iNOS in both unexposed and SM-exposed SAE and BTE cells.

The reduced overexpression of proinflammatory cytokines and iNOS by roxithromycin in SM-exposed SAE and BTE cells is in agreement with previous *in vivo *and *in vitro *studies (for a complete review see [27]) and further supports the contention that macrolides can inhibit the production of proinflammatory mediators and cytokines. This suppressive effect of roxithromycin may not be accounted for by its antimicrobial properties but rather by its anti-inflammatory actions, as evidenced by the fact that other antibiotics, such as amoxicillin, cefaclor, penicillin, and cephalosporin, had no such effects even at high concentration (data not shown). Thus the immunomodulatory effects of antibiotics appear to be specific to macrolides. Similar findings were made by Kohri *et al*. [28].

The protective effect of roxithromycin on SM cytotoxicity could be explained, at least in part, by its ability to inhibit the overexpression of pro-inflammatory cytokines and mediators. As mentioned previously, Qabar *et al*. [20] reported that suppressed expression of the proinflammatory cytokines IL-8 and IL-6 in human epidermal keratinocytes, mediated by overexpressing the anti-inflammatory cytokine IL-10, led to increased viability of SM-treated cells. Also, 1-alpha, 25-dihydroxyvitamin D3 enhanced cell proliferation in human skin cells stimulated with SM by suppressing expression of the inflammatory mediators IL-6 and IL-8 [29]. On the other hand, Stone *et al*. [30] reported that inflammatory cytokines, such as TNF-α and IL-1β enhanced SM toxicity on murine macrophages. The protective effect of roxithromycin seen in this study, an increase in cell viability by ~2.5 and ~3.0 times for SAE and BTE cells respectively, is higher than that of 1-alpha, 25-dihydroxyvitamin D3 on HEK cells, an increase of ~1.5 times in cell proliferation [29]; although the results are not directly comparable as the cells used in the two studies are different.

The subcellular mechanism of the anti-inflammatory effect of macrolides remains unknown. However, it is well known that the expression of several genes involved in the immune and inflammatory response (e.g., iNOS, TNF, IL-1, IL-6, IL-8) are regulated at the transcriptional level by nuclear factor-κB (NF-κB) (for a recent review, see [31]). Thus, it is conceivable that macrolide antibiotics may act as anti-inflammatory agents by preventing the activation of NF-κB. Ichiyama *et al*. [32] demonstrated that clarithromycin, another macrolide widely used clinically, inhibits NF-κB-dependent reporter gene expression in transfected pulmonary epithelial cells, providing direct evidence in support of specific effects of macrolides on NF-κB activation. However, it is now held that the molecular mechanism(s) by which macrolides inhibit pro-inflammatory cytokine responses in the respiratory epithelium varies depending upon the macrolide used, the activating stimulus, and the cell type examined [27]. Thus, further studies are required to determine whether NF-κB is the target molecule for roxithromycin in the signal transduction pathway in SM-exposed SAE and BTE cells.

## Conclusion

Our results demonstrate that roxithromycin has anti-cytotoxic and anti-inflammatory activities in human airway epithelial cells. These effects of roxithromycin likely depend on its ability to prevent the overproduction of proinflammatory cytokines and mediators. Thus, macrolide antibiotics can exert therapeutic effects independent of their antibacterial activity. In view that there are currently no effective antidotes for SM inhalation injuries, the results of the present study suggest that macrolide antibiotics may serve as potential vesicant respiratory therapeutics.

## Methods

### Reagents

Sulfur mustard (2,2'-dichlorodiethyl sulfide; 4 mM) was acquired from the US Army Edgewood Research, Development and Engineering Center (Aberdeen Proving Ground, MD). Roxithromycin was obtained from Sigma (St. Louis, MO) and dissolved in ethanol at a concentration of 10 mM and then diluted to the desired concentrations using culture medium.

### Cell culture

Normal human SAE cells and normal human BTE cells were obtained from Cambrex (Walkersville, MD). The homogeneity of these cells was vigorously tested and assured by the manufacturer. Cells were grown in the optimized media as formulated by the manufacturer and cultured at 37°C under humidified 5% CO_2_. Experiments were performed on cells of the fourth passage. For all experiments, cells were grown to ~90% confluence except for the immunofluorescence staining experiment, where cell density was 30 – 40% confluence.

### Sulfur mustard exposure

Cells were seeded in 6-, 12-, 24- or 96-well plates and allowed to grow for 5 to 6 days to near confluence. The medium was replaced with fresh medium before exposure. SM was then added to the final concentrations indicated. The culture plates were maintained at room temperature in a chemical fume hood for 1 h then transferred to an incubator of 37°C with 5% CO_2_.

### Cytotoxicity assay

Cytotoxicity was measured by either the MTS assay [33] or Calcein AM/ethidium homodimer-1 (EthD-1) staining [19]. The CellTiter 96 Aqueous One Solution Cell Proliferation Assay kit from Promega (Madison, WI) was used for the MTS assay and instructions from the manufacturer were followed. Briefly, cells were seeded in a 96-well plate at 1 × 10^4 ^cells/well and allowed to adhere overnight at 37°C with 5% CO_2_, then cells were exposed to SM with or without roxithromycin and incubated for another 24 h. After adding 20 μl of assay reagent to each well that contained 100 μl medium, cells were further cultured for 3 h and the resultant absorbance was recorded at 490 nm using a 96-well plate reader. Each experiment was performed with eight independent replicates and repeated three times. For the Calcein AM/EthD-1 staining method, the LIVE/DEAD Viability/Cytotoxicity Kit from Molecular Probes (Eugene, OR) was used and protocols provided by the manufacturer were adopted.

### Enzyme-Linked Immunosorbent Assay (ELISA)

ELISA kits from BD Biosciences (San Diego, CA) were used for the quantification of IL-1β, IL-6, IL-8, and TNF in the culture medium following the manufacturer's instructions. The results are expressed as a percentage of the control (cells unexposed to SM) and represent the mean ± SE of three experiments performed in triplicate.

### Real-Time RT-PCR

Total RNA was isolated from ~1 × 10^7 ^cells of each sample, using the RNAqueous-4PCR kit from Ambion (Austin, TX) following the manufacturer's instructions and with the optional DNase I treatment step to avoid contamination with genomic DNA. Reverse transcription of mRNA was carried out using the High-Capacity cDNA Archive Kit from Applied Biosystems (Foster City, CA), using 1 μg of total RNA in an 100 μl final volume. Real-time PCR was carried out on the 7500 Fast Real-Time PCR system of Applied Biosystems using TaqMan Gene Expression Assays primer/probe sets and the standard thermal-cycling conditions for relative quantification designed by the manufacturer. Results were analyzed with the SDS software v1.3.0 on the system using the 2−ΔΔCT
 MathType@MTEF@5@5@+=feaafiart1ev1aaatCvAUfKttLearuWrP9MDH5MBPbIqV92AaeXatLxBI9gBaebbnrfifHhDYfgasaacH8akY=wiFfYdH8Gipec8Eeeu0xXdbba9frFj0=OqFfea0dXdd9vqai=hGuQ8kuc9pgc9s8qqaq=dirpe0xb9q8qiLsFr0=vr0=vr0dc8meaabaqaciaacaGaaeqabaqabeGadaaakeaacqaIYaGmdaahaaWcbeqaaiabgkHiTiabgs5aejabgs5aeHqaaiab=neadnaaBaaameaacqWFubavaeqaaaaaaaa@33F4@ method. β-actin was used as an endogenous control to correct for variations in input RNA amount and cDNA amplification of different samples.

### Immunofluorescence staining

Cells were grown on slide to 30% – 40% confluence and exposed to SM with or without roxithromycin. After 24 h, cells were rinsed with 10 mM Tris, 150 mM NaCl, pH 7.4 (TBS) and fixed in 10% zinc formalin solution. The slides were then processed robotically on the BenchMark XT IHC/ISH workstation of Ventana (Tucson, AZ) to react with the primary and secondary antibodies. The primary antibody used was mouse anti-iNOS MAb from BD Biosciences, and was diluted 1:1000 in TBS + 0.5% (w/v) Triton X-100 (TBST). The secondary antibody was pre-diluted biotinylated rabbit anti-mouse IgG from Ventana. iNOS signals were detected by quantum dot-streptavidin conjugates (streptavidin-Qdot655), which was diluted 1:200 with the Qdots incubation buffer from Invitrogen (Carlsbad, CA). The fluorescence signals were visualized, captured and processed as reported previously [25,26].

### Data analysis

For comparative studies, Student's *t*-test (unpaired) or one-way ANOVA tests (with Bonferroni post test if *P *< 0.05) were used for statistical analysis. Differences were considered statistically significant if a *P *value of < 0.05 was achieved.

## Abbreviations

BTE, bronchial/tracheal epithelial

calcein AM, calcein acetoxy methyl ester

ELISA, enzyme-linked immunosorbent assay

EthD-1, ethidium homodimer-1

IL, interleukin

iNOS, inducible nitric oxide synthase

MTS, 3-(4,5-dimethylthiazol-2-yl)-5-(3-carboxymethoxyphenyl)-2-(4-sulfophenyl)-2H-tetrazolium, inner salt

NO, nitric oxide

RT-PCR, reverse transcriptase polymerase chain reaction

NOS, nitric oxide synthase; RXM, roxithromycin

SAE, small airway epithelial; SE, standard error

SM, sulfur mustard

TNF, tumor necrosis factor

TBS, tris-buffered saline

TBST, tris-buffered saline with Triton.

## Authors' contributions

PR originated the project, supervised the overall conduct of the research which was performed in her laboratory, and provided continuous evaluation of the experimental data. XG carried out all of the experimental work in this study, performed the statistical analyses, and drafted the manuscript. RR (along with PR) conceived of the study and carried out SM exposure experiment. YX and PEB carried out the immunocytochemical studies and analyzed the data. All authors read and approved the final manuscript.
